# Trends in antimicrobial resistance and antibiotic use before and during the coronavirus disease 2019 pandemic in a university research and practice hospital in Türkiye

**DOI:** 10.1099/acmi.0.001150.v4

**Published:** 2026-03-19

**Authors:** Merve Buyukcelik, Fusun Zeynep Akcam, Ersin Uskun, Emel Sesli Cetin, Onur Kaya, Gul Ruhsar Yilmaz, Onur Unal, Esra Nurlu Temel

**Affiliations:** 1 Department of Infectious Diseases and Clinical Microbiology, Suleyman Demirel University Medical School, Isparta, Türkiye; 2 Department of Public Health, University Medical School, Isparta, Türkiye; 3 Department of Microbiology, University Medical School, Isparta, Türkiye

**Keywords:** antibacterial drugs, antimicrobial resistance, antimicrobial stewardship, carbapenem resistance, extended-spectrum *β*-lactamase (ESBL), pandemic

## Abstract

**Introduction.** Antibacterial resistance and the increasing number of infections caused by multidrug-resistant bacteria threaten human health worldwide. The coronavirus disease 2019 (COVID-19) pandemic may have influenced antibacterial resistance patterns through changes in infection control practices and antibiotic prescribing.

**Aim.** This single-centre, retrospective study aimed to describe changes in bacterial distribution, antibacterial resistance and antibiotic consumption in a university research and practice hospital before and during the COVID-19 pandemic.

**Methods.** We analysed routine microbiology and pharmacy records from hospitalized patients between 01 April 2018 and 31 March 2022. The 2 years before 01 April 2020 was defined as the pre-pandemic period and the 2 years after 01 April 2020 as the pandemic period. Bacteria isolated from blood, urine and lower respiratory tract cultures, together with their antimicrobial susceptibility profiles, were compared between periods according to EUCAST criteria. Antibiotic consumption was calculated as defined daily doses per 1,000 inpatient days for commonly used agents. No patient-level clinical data or ward/ICU stratification was available.

**Results.** A total of 7,275 isolates from 47,729 culture samples were obtained in the pre-pandemic period and 5,794 isolates from 47,210 samples during the pandemic. Coagulase-negative staphylococci remained the most frequently isolated species from blood cultures, *Escherichia coli* from urine cultures and *Acinetobacter baumannii* from lower respiratory tract cultures in both periods. Extended-spectrum *β*-lactamase (ESBL) rates and carbapenem resistance in *E. coli* and *Klebsiella pneumoniae* increased significantly during the pandemic, whereas teicoplanin and linezolid resistance in coagulase-negative staphylococci decreased. Carbapenem resistance in *A. baumannii* also decreased. Overall antibiotic consumption increased for most agents, particularly cephalosporins, carbapenems, aminoglycosides and fluoroquinolones, while vancomycin use decreased.

**Conclusion.** In this single-centre, retrospective analysis, the overall distribution of major bacterial species remained largely stable before and during the COVID-19 pandemic, whereas important changes were observed in antimicrobial resistance profiles and antibiotic consumption. The increase in ESBL and carbapenem resistance in *Enterobacterales*, together with higher use of broad-spectrum antibiotics, underlines the need for strengthened antimicrobial stewardship and continuous local surveillance.

## Data Summary

All data associated with this work is reported within the article.

## Introduction

Antimicrobial resistance (AMR) is recognized as one of the major global public health threats of the 21st century. The increasing prevalence of infections caused by multidrug-resistant (MDR) Gram-negative bacilli and resistant Gram-positive cocci is associated with higher morbidity, mortality, length of hospital stay and healthcare costs [1]. Hospitals, especially tertiary care centres, represent key settings both for the emergence of MDR organisms and for the implementation of effective antimicrobial stewardship and infection prevention strategies.

The coronavirus disease 2019 (COVID-19) pandemic has profoundly affected hospital organization, patient case mix and clinical practice. Changes in patient referral patterns, expansion of intensive care capacity, staff redeployment, use of personal protective equipment and modifications in infection control procedures have all had the potential to influence hospital epidemiology. In addition, high rates of empirical antibacterial prescribing during the early phases of the pandemic, often in the absence of proven bacterial co-infection, raised concerns about a possible acceleration of AMR [2]. However, published studies evaluating the impact of COVID-19 on AMR and antibiotic use at the hospital level have reported heterogeneous results, with some institutions observing increased rates of certain MDR pathogens and others reporting stable or even decreased resistance trends.

Data from Türkiye regarding hospital-level changes in bacterial epidemiology, resistance patterns and antibiotic consumption during the COVID-19 period are still limited, particularly from university research and practice hospitals. A better understanding of resistance trends is essential for guiding empirical therapy, optimizing antimicrobial stewardship interventions and supporting infection control policies. Therefore, this study aimed to compare the distribution of bacterial isolates, their antimicrobial susceptibility profiles and antibiotic consumption indices in our tertiary care university hospital during the 2 years before and the 2 years after the onset of the COVID-19 pandemic.

## Methods

In this study, the distribution of bacteria, antibiotic susceptibilities and antibiotic consumption obtained from blood, urine and lower respiratory tract (LRT) samples of patients who were followed and treated as inpatients at Süleyman Demirel University Research and Application Hospital, a 700-bed tertiary general hospital, between 01 April 2018 and 31 March 2022 were retrospectively analysed. The 2 years before 01 April 2020 was defined as the pre-pandemic period, and the 2 years after 01 April 2020 was defined as the pandemic period. Data from both periods were statistically compared with each other.

All bacteria isolated from clinical samples within the study period and antibiotic susceptibilities were obtained from the hospital bacteriology laboratory, and antibiotic information was obtained from hospital pharmacy records. Culture results of outpatients and recurrent growths from the same patient within 10 days were excluded from the study.

Approval for the study was obtained from the local ethics committee.

All relevant data are included in the article and its supplementary material. Additional data supporting the findings of this study are available also from the corresponding author upon reasonable request and with permission of the institution.

### Bacterial identification and antibiotic susceptibility tests

Identification of bacteria was performed using conventional methods and the BD Phoenix 100 (BectonDickinson, NJ, USA) automated system. Antimicrobial susceptibility was assessed using the disc diffusion method, the gradient strip test (E-test) and an automated antibiotic susceptibility testing system. Antibiotic susceptibility was based on zone diameters and MIC values published in the European Committee on Antimicrobial Susceptibility Testing (EUCAST) guidelines. Antibiotic susceptibility for each culture was based on zone diameters and MIC values published in the current EUCAST guidelines for the testing year. Extended-spectrum *β*-lactamase (ESBL) detection was performed by a combined disc test and the BD Phoenix 100 automated system.

Antibiotic susceptibilities of the isolates were evaluated according to EUCAST criteria. In line with EUCAST recommendations for serious infections, isolates categorized as ‘susceptible, increased exposure’ (formerly ‘intermediate’) were grouped together with susceptible isolates for the purposes of analysis, as our objective was to describe temporal trends rather than to estimate precise clinical breakpoints. Tigecycline could not be evaluated in our study because it was not included in the ready panels uploaded to BD Phoenix for some years. Colistin susceptibility was evaluated using the liquid microdilution method, the reference standard for determining colistin antimicrobial susceptibility in the relevant isolates.

The antimicrobial susceptibility of *Enterococcus faecium*, *Enterococcus faecalis*, *Staphylococcus aureus* and coagulase-negative staphylococci (CoNS) isolates found to be resistant to teicoplanin, vancomycin and linezolid by automated system results was confirmed by the gradient strip test method.

A Gram-negative isolate resistant to any carbapenem was considered carbapenem resistant.

### Calculation of antibiotic consumption

Antibiotic consumption indices were calculated by standardizing the consumption of cefazolin, ceftriaxone, ampicillin–sulbactam, piperacillin–tazobactam, meropenem, imipenem, vancomycin, teicoplanin, amikacin, gentamicin, ciprofloxacin and levofloxacin according to the defined daily dose (DDD) per 1,000 inpatient days for each antibiotic determined by the WHO. Inpatient days were obtained from the database of the hospital data processing unit.

### Statistical analysis

The data were evaluated using the computerized program SPSS.26 (International Business Machines Corporation, NY, USA). Number and percentage values were used as descriptive statistics, and the chi-square test was used in analytical evaluations. In all analyses*, P<*0.05 was accepted as the cut-off value for statistical significance. The findings of the statistical analysis were presented with the exact *P*-values. When the value was less than 0.001, it was expressed as *P<*0.001. Relative increase was calculated by dividing the absolute difference between periods by the pre-pandemic rate and expressed as a percentage.

### Study design considerations

This investigation was designed as a single-centre, retrospective, laboratory- and pharmacy-based surveillance study. Only routine microbiology data and aggregate antibiotic consumption indices were available; no patient-level clinical data (such as age, comorbidities, COVID-19 status or outcomes) or stratification by ward type or intensive care unit was accessible. Repeated isolates from the same patient collected more than 10 days apart were considered separate episodes, which may overestimate resistance rates in patients with prolonged hospital stays. Antibiotic consumption was expressed as DDDs per 1,000 inpatient days without adjustment for changes in case mix or hospital occupancy over time. Therefore, our findings should be interpreted as descriptive ecological trends rather than as evidence of causal relationships between antibiotic use, infection control measures and resistance patterns.

## Results

A total of 7,275 bacteria were isolated from 47,729 blood, urine and LRT culture samples taken in the pre-pandemic period. During the pandemic period, 5,794 bacteria were isolated from 47,210 such samples. Thus, the number of processed cultures remained broadly similar, while the total number of isolates decreased. The distribution of isolates by specimen type and study period is presented in Table 1.

**Table 1.** Number of isolates obtained from culture samples

	Blood	Urine	LRT	Total
Pre-pandemic period	3,648	2,512	1,115	7,275
During pandemic	2,970	2,005	819	5,794
Total	6,618	4,517	1,934	13,069

In both periods, CoNS remained the most frequently isolated organisms from blood cultures, accounting for ~63.6% (2,319/3,648) and 65.5% (1,946/2,970) of blood isolates in the pre-pandemic and pandemic periods, respectively. *Escherichia coli* continued to predominate in urine cultures, representing 39.3% (986/2,512) and 38.7% (776/2,005) of isolates, while *Acinetobacter baumannii* was the leading pathogen in LRT cultures [28.5% (318/1,115) and 29.1% (238/819) of isolates in the two periods]. The distribution of the ten most frequently isolated micro-organisms by specimen type and study period is presented in Table 2. Given that CoNS are frequent skin contaminants, the very high proportion of CoNS among blood culture isolates likely reflects a mixture of true bloodstream infections and culture contamination and should therefore be interpreted with caution.

**Table 2.** The most frequently isolated micro-organisms from clinical specimens

Sample	Pre-pandemic period		During pandemic	
	Bacteria	% (*n*)	Bacteria	% (*n*)
Blood	Total	100.0 (3,648)	Total	100.0 (2,970)
	CoNS	63.6 (2,319)	CoNS	65.5 (1,946)
	*E. coli*	5.1 (185)	*E. faecium*	7.1 (212)
	*E. faecium*	3.8 (137)	*E. coli*	4.7 (141)
	*A. baumannii*	3.7 (134)	*K. pneumoniae*	3.6 (107)
	*S. aureus*	3.6 (130)	*S. aureus*	3.4 (101)
	*Streptococcus* spp.	3.3 (121)	*A. baumannii*	3.3 (98)
	*E. faecalis*	2.6 (95)	*E. faecalis*	2.4 (72)
	*K. pneumoniae*	2.5 (91)	*P. aeruginosa*	2.2 (65)
	*P. aeruginosa*	1.6 (59)	*Streptococcus* spp.	2.0 (58)
	*E. cloacae*	1.0 (38)	*Corynebacterium* spp.	0.9 (28)
Urine	Total	100.0 (2,512)	Total	100.0 (2,005)
	*E. coli*	39.3 (986)	*E. coli*	38.7 (776)
	*K. pneumoniae*	12.8 (321)	*E. faecium*	15.0 (301)
	*E. faecium*	10.0 (251)	*K. pneumoniae*	13.6 (273)
	*E. faecalis*	8.0 (200)	*E. faecalis*	7.2 (144)
	*P. aeruginosa*	7.2 (181)	*P. aeruginosa*	6.5 (130)
	CoNS	3.9 (98)	*A. baumannii*	4.9 (99)
	*A. baumannii*	3.8 (95)	CoNS	3.2 (64)
	*P. mirabilis*	3.1 (77)	*P. mirabilis*	3.1 (62)
	*Enterococcus* spp.	1.6 (40)	*K. oxytoca*	0.9 (18)
	*E. cloacae*	1.5 (38)	*E. cloacae*	0.8 (17)
LRT	Total	100.0 (1,115)	Total	100.0 (819)
	*A. baumannii*	28.5 (318)	*A. baumannii*	29.1 (238)
	*P. aeruginosa*	16.5 (184)	*K. pneumoniae*	13.1 (107)
	*E. coli*	8.4 (94)	*P. aeruginosa*	12.7 (104)
	*K. pneumoniae*	8.4 (94)	*E. coli*	12.5 (102)
	*S. aureus*	7.8 (87)	*S. maltophilia*	8.5 (70)
	*S. maltophilia*	6.3 (70)	*S. aureus*	5.6 (46)
	*E. cloacae*	2.2 (24)	CoNS	3.7 (30)
	*K. oxytoca*	1.7 (19)	*E. faecium*	2.1 (17)
	*Acinetobacter* spp.	1.6 (18)	*Acinetobacter* spp.	1.8 (15)
	Nonfermenting gram-negative bacilli	1.6 (18)	*E. cloacae*	1.6 (13)

LRT, lower respiratory tract.

Oxacillin and teicoplanin resistance in CoNS isolates decreased statistically significantly during the pandemic period, whereas there was no significant difference between the periods in the oxacillin resistance of *S. aureus* isolates. Vancomycin resistance was not observed in staphylococci in either period. Antibiotic resistance results of the most frequently isolated Gram-positive bacteria are presented in Table 3. During the pandemic period, there was a statistically significant increase in ESBL rates of *E. coli* and *Klebsiella pneumoniae* strains. The ESBL frequency of *E. coli* increased from 43.7% before the pandemic to 49.3% during the pandemic (an absolute increase of 5.6% points, corresponding to a relative increase of ~13%), while that of *K. pneumoniae* rose from 58.9 to 67.9% (an absolute increase of 9.0% points, corresponding to a relative increase of ~15%). Carbapenem resistance in *E. coli* and *K. pneumoniae* also increased significantly, whereas carbapenem resistance in *A. baumannii* decreased (Table 4). These changes are clinically important, as they further restrict options for empirical and targeted therapy in serious infections caused by these pathogens. Overall antibiotic resistance patterns and carbapenem resistance rates of the most frequently isolated Gram-negative bacteria are summarized in Table 4.

**Table 3.** Resistance of Gram-positive bacteria to various antibiotics

Antibiotic	CoNS			*S. aureus*			*E. faecalis*			*E. faecium*		
	Pre-pandemic	During pandemic	*P*-value	Pre-pandemic	During pandemic	*P*-value	Pre-pandemic	During pandemic	*P*-value	Pre-pandemic	During pandemic	*P*-value
AMP	–	–	–	–	–	–	8.0	6.8	0.621	88.3	83.2	0.028
OXA	78.0	72.2	**<0.001**	19.7	26.1	0.136	–	–	–	–	–	–
TEI	1.4	0.5	**0.003**	1.4	0.7	0.648	1.2	3.1	0.188	22.9	11.0	**<0.001**
VAN	0.0	0.0	–	0.0	0.0	–	1.0	1.4	0.704	20.7	9.9	**<0.001**
LZD	3.0	0.2	**<0.001**	0.0	0.0	–	0.0	0.0	–	0.8	0.4	0.658
DAP	3.5	4.0	0.514	1.3	4.9	0.124	–	–	–	–	–	–
SXT	30.0	39.0	**<0.001**	3.4	10.3	**0.005**	–	–	–	–	–	

Statistically significant p-values are written in bold. Sensitivity results that were not given a value were either not tested or were omitted because they were not significant.

AMP, ampicillin; DAP, daptomycin; LZD, linezolid; OXA, oxacillin; SXT, trimethoprim sulfamethoxazole; TEI, teicoplanin ; VAN, vancomycin.

**Table 4.** Resistance of Gram-negative bacteria to various antibiotics and ESBL and carbapenem resistance rates

	*E. coli*			*K. pneumoniae*			*P. aeruginosa*			*A. baumannii*		
Antibiotic	Pre-Pandemic	Pandemic	*P*-value	Pre-Pandemic	Pandemic	*P*-value	Pre-Pandemic	Pandemic	*P*-value	Pre-Pandemic	Pandemic	*P*-value
CRO	43.4	45.5	0.344	58.3	65.4	**0.025**	–	–	–	–	–	–
CAZ	45.3	54.9	**<0.001**	61.0	72.7	**<0.001**	24.6	21.0	0.267	93.5	91.3	0.417
ETP	8.6	13.5	**<0.001**	40.2	52.8	**<0.001**	–	–	–	–	–	–
MEM	2.7	5.0	**0.005**	22.9	44.1	**<0.001**	31.0	27.8	0.354	91.0	82.1	**<0.001**
IMP	1.2	4.9	**<0.001**	19.0	43.5	**<0.001**	34.3	35.9	0.653	91.1	85.0	**0.003**
SXT	45.7	40.0	**0.006**	46.1	52.3	0.052	–	–	–	76.8	75.6	0.655
AK	1.6	3.1	**0.014**	10.4	18.1	**0.001**	5.4	7.2	0.342	79.7	76.0	0.167
COL	44.4	16.7	0.580	64.1	52.8	0.140	11.8	4.3	0.565	14.2	16.9	0.353
ESBL	43.7	49.3	**0.021**	58.9	67.9	**0.004**	–	–	–	–	–	–
Carbapenem resistance	8.6	14.0	**<0.001**	39.9	52.4	**<0.001**	37.3	40.8	0.336	90.3	83.7	**0.002**

Statistically significant p-values are written in bold. Sensitivity results that were not given a value were either not tested or were omitted because they were not significant.

AK, amikacin; CAZ, ceftazidime; COL, colistin; CRO, ceftriaxone; ETP, ertapenem; IMP, imipenem; MEM, meropenem; SXT, trimetoprim sulfamethoxazole.

In both periods, cephalosporins were the most frequently prescribed, followed by penicillins and carbapenems. Antibiotic consumption indices increased for all studied agents except vancomycin during the pandemic period (Table 5). The most pronounced rises were observed for teicoplanin (from 21.24 to 54.81 DDD per 1,000 inpatient days) and levofloxacin (from 48.81 to 119.78 DDD per 1,000 inpatient days). Moderate increases were also seen for third-generation cephalosporins, carbapenems, aminoglycosides and fluoroquinolones. These findings indicate a broader shift towards greater use of broad-spectrum antibiotics during the pandemic period.

**Table 5.** Antibiotic consumption indices

Antibiotic	Antibiotic consumption	
	DDD/1,000 patient days	
	**Pre-pandemic**	**Pandemic**
Cefazolin	83.48	119.81
Ceftriaxone	125.67	141.01
Ampicillin–sulbactam	68.49	93.48
Piperacillin–tazobactam	64.98	68.46
Meropenem	68.55	73.19
Imipenem	5.72	7.28
Vancomycin	13.48	10.35
Teicoplanin	21.24	54.81
Amikacin	21.73	25.68
Gentamicin	9.88	30.82
Ciprofloxacin	27.08	37.09
Levofloxacin	48.81	119.78

DDD, defined daily dose.

## Discussion

Antibacterial resistance, along with the rising prevalence of infections caused by MDR bacteria, poses a significant threat to global human health [3]. The COVID-19 pandemic encompasses factors that could either exacerbate or mitigate antibacterial resistance. This study provides a 4-year overview of bacterial epidemiology, AMR patterns and antibiotic consumption in a tertiary university research and practice hospital spanning the 2 years before and the 2 years during the COVID-19 pandemic. Overall, the distribution of the main bacterial species isolated from blood, urine and LRT cultures remained broadly stable across the study periods, with CoNS dominating blood cultures, *E. coli* predominating in urine cultures and *A. baumannii* remaining the leading pathogen in LRT cultures (Table 2). In the study conducted by Bozok and Öztürk [4], findings similar to ours were reported. However, in contrast to this stability, important changes were observed in resistance profiles, including increasing ESBL production and carbapenem resistance among *E. coli* and *K. pneumoniae* and decreasing carbapenem resistance in *A. baumannii*, together with substantial rises in the consumption of several broad-spectrum antibiotics.

Our findings are largely consistent with previous hospital-based studies that have described the burden of MDR Gram-negative bacilli and resistant Gram-positive cocci in both pre-pandemic and pandemic periods [4,9]. In recent years, Gram-positive bacteria, mostly staphylococci, have been isolated most frequently from blood cultures [10,11]. The very high proportion of CoNS among blood culture isolates in our study also mirrors observations from other institutions [4,6,12]. However, because CoNS are common skin commensals and frequent contaminants of blood cultures, it is likely that our data reflect a mixture of true bloodstream infections and contamination events. Without patient-level clinical information, we were not able to distinguish between these scenarios, and our CoNS findings should therefore be interpreted with caution.

Gram-negative pathogens are more frequently isolated in hospital-acquired pneumonia compared to community-acquired pneumonia [13]. In our study, pathogens associated with nosocomial pneumonia were frequently isolated from LRT culture samples in both periods. This likely reflects local care pathways, whereby patients with community-acquired pneumonia are mainly managed in outpatient settings, while our analysis focused on cultures obtained from hospitalized patients.

The observed increases in ESBL production and carbapenem resistance among *E. coli* and *K. pneumoniae* during the pandemic period are concerning, as they further limit options for empirical and targeted therapy in serious infections. Several studies from different regions have reported similar trends in *Enterobacterales*, sometimes temporally associated with increased use of broad-spectrum antibiotics, high patient acuity and strained infection prevention resources during COVID-19 surges [12,14,17]. In our hospital, the rise in ESBL and carbapenem resistance coincided with greater use of third-generation cephalosporins, carbapenems and fluoroquinolones. Although this temporal association is biologically plausible, the ecological design of our study does not allow us to infer a direct causal relationship between antibiotic consumption and resistance. Other unmeasured factors, such as changes in patient populations, transfer patterns from other hospitals or undetected clonal outbreaks, may also have contributed.

Conversely, we also observed a decrease in resistance to some agents in certain bacterial species. Carbapenem resistance was reduced in *A. baumannii*, teicoplanin and linezolid resistance in CoNS was reduced and vancomycin and teicoplanin resistance was reduced in *E. faecium*. Divergent trends in different pathogen–antibiotic combinations have also been reported in other hospital-level analyses conducted during the COVID-19 era [4,9, 12, 18,20]. Several potential explanations can be considered. Intensified infection prevention and control measures, including universal masking, enhanced hand hygiene and cohorting of patients, may have contributed to the observed reductions. Reorganization of wards and intensive care units, with periods of reduced elective activity, might also have altered opportunities for transmission. In addition, local changes in empirical therapy protocols or restricted use of certain agents could have exerted selective pressure in favour of more susceptible subpopulations. However, these hypotheses remain speculative in the absence of detailed data on infection control interventions, ward-level activity and molecular typing.

Our analysis of antibiotic consumption showed that DDDs per 1,000 inpatient days increased for all studied agents except vancomycin during the pandemic period, with the largest relative increases observed for teicoplanin and levofloxacin. This pattern is broadly in line with reports from other hospitals describing increased empirical antibiotic use in patients with suspected or confirmed COVID-19, particularly during the early phases of the pandemic when concerns about bacterial co-infections were high and robust evidence on their true frequency was still emerging [7,8, 14, 21, 22]. In our setting, the rise in broad-spectrum antibiotic consumption should be interpreted in the context of changes in hospital activity and case mix, which we were unable to quantify. We cannot determine to what extent the observed increases represent appropriate treatment of more severe or complex infections versus potentially avoidable prescribing.

Our study has several strengths. It covers a relatively long period that includes two full years before and after the onset of the COVID-19 pandemic, allowing trends rather than short-term fluctuations to be examined. It integrates routine microbiology data with hospital-wide antibiotic consumption indices, providing a comprehensive picture of local AMR dynamics and prescribing patterns. Furthermore, it contributes to the still limited body of data from Türkiye on hospital-level changes in AMR and antibiotic use associated with the COVID-19 period, particularly from university research and practice hospitals. Such locally generated evidence is essential to inform empirical therapy choices, to support targeted antimicrobial stewardship strategies and to guide infection prevention policies.

The limitations of our study should be acknowledged. First, its retrospective, single-centre design based on routine laboratory and pharmacy records limits the generalizability of the findings and precludes adjustment for patient-level factors such as age, comorbidities, COVID-19 status or illness severity. Second, we were not able to distinguish colonization from true infection, and the very high proportion of CoNS in blood cultures suggests that contamination may have contributed to some of the observed patterns. Third, repeated isolates from the same patient more than 10 days apart were counted as separate episodes, which could overestimate resistance rates in patients with prolonged admissions. Fourth, we did not have information on ward type or intensive care unit versus general ward location, nor on changes in hospital activity, case mix or admission numbers during the pandemic period. Finally, antibiotic consumption was analysed as aggregate DDD per 1,000 inpatient days without risk adjustment. Taken together, these limitations mean that our results should be interpreted as descriptive ecological trends rather than as proof of direct causal effects of the COVID-19 pandemic or of antibiotic consumption on resistance.

In conclusion, the COVID-19 pandemic period in our hospital was characterized by largely stable distributions of the main bacterial species isolated from blood, urine and LRT cultures, but by important shifts in AMR profiles and antibiotic consumption. ESBL production and carbapenem resistance increased in *E. coli* and *K. pneumoniae*, whereas carbapenem resistance in *A. baumannii* and resistance to certain agents in Gram-positive cocci decreased. At the same time, the use of several broad-spectrum antibiotics increased, with the exception of vancomycin. Although the ecological nature of our analysis does not allow causal inferences, these findings underscore the importance of continuous local surveillance and adaptive antimicrobial stewardship programmes that can adapt to changing clinical and organizational conditions during and beyond pandemics.

## Supplementary material

10.1099/acmi.0.001150.v4 Supplementary Material 1.

## Abbreviations

AMR: antimicrobial resistance
CoNS: coagulase-negative staphylococci
COVID-19: coronavirus disease 2019
DDD: defined daily dose
EUCAST: European Committee on Antimicrobial Susceptibility Testing
ESBL: extended-spectrum *β*-lactamase
LRT: lower respiratory tract
MDR: multidrug-resistant
